# Task Engagement in Matrix Reasoning Performance: A Cross-Cultural Investigation in China and the United Kingdom

**DOI:** 10.3390/jintelligence14070117

**Published:** 2026-06-25

**Authors:** Rui Wang, Kastoori Kalaivanan, Jiani Ren, Shen-Hsing Annabel Chen, Chew Lee Teo

**Affiliations:** 1 Centre for Research and Development in Learning (CRADLE), Nanyang Technological University, 50 Nanyang Avenue, Singapore 639798, Singapore; rui.wang@ntu.edu.sg (R.W.); kastoori.k@ntu.edu.sg (K.K.); jiani.ren@ntu.edu.sg (J.R.); annabelchen@ntu.edu.sg (S.-H.A.C.); 2 Faculty of Education, University of Cambridge, 184 Hills Road, Cambridge CB2 8PQ, UK; 3 National Institute of Education, Nanyang Technological University, 1 Nanyang Walk, Singapore 637616, Singapore; 4 Psychology, School of Social Sciences, Nanyang Technological University, 48 Nanyang Avenue, Singapore 639818, Singapore; 5 Lee Kong Chian School of Medicine (LKCMedicine), Nanyang Technological University, 11 Mandalay Road, Singapore 308232, Singapore

**Keywords:** fluid intelligence, matrix reasoning, cross-cultural cognition, response time, task engagement, item response theory

## Abstract

Matrix reasoning tasks remain among the most widely used instruments for assessing abstract reasoning and are often assumed to be culturally neutral. However, this assumption has been challenged by studies reporting significant cross-cultural variation in performance on nonverbal matrix reasoning tasks, even when groups show comparable performance on verbal measures of general cognitive ability. One plausible reason is that many matrix reasoning tasks rely primarily on accuracy-based performance metrics while providing limited insight into response timing and task engagement during problem solving. The present study examined the Matrix Reasoning Item Bank (MaRs-IB), a new online matrix reasoning instrument integrating both accuracy and response time, in 458 participants from China and the UK. Results demonstrated strong psychometric properties across both cultural contexts, while also revealing systematic between-group differences in overall task performance. Chinese participants were generally slower but more accurate, whereas UK participants responded more quickly with lower overall accuracy. Rather than reflecting a classical speed–accuracy trade-off, these patterns may indicate cross-cultural variation in persistence, deliberative engagement, and the metacognitive regulation of cognitive effort during reasoning tasks. In particular, Chinese participants allocated more time before responding and persisted longer on challenging task items, whereas UK participants demonstrated relatively faster responding and shorter response times on more challenging items. These findings suggest that cross-cultural differences in matrix reasoning performance may reflect not only differences in observed performance levels, but also variation in how participants allocate time and sustain engagement during cognitively demanding tasks.

## 1. Introduction

Abstract reasoning has long been conceptualized as a multifaceted construct encompassing the ability to reason, solve problems, and think abstractly without relying on task-specific knowledge (Brydges et al., 2012; Burkart et al., 2017; Conway et al., 2003; Ellefson et al., 2020; Kane et al., 2004). Abstract reasoning is recognized as a core aspect of fluid intelligence (Perfetti et al., 2009) and is closely associated with non-verbal reasoning abilities (Chouinard-Gaouette & Blanchette, 2024). Matrix reasoning tasks are among the most commonly used instruments for assessing non-verbal abstract reasoning across cultural contexts, yet their cultural fairness has increasingly been questioned due to methodological features and task demands that may not be entirely culturally neutral (Gonthier, 2022). One possible contributor to cross-cultural variation in matrix reasoning performance is that most existing measures rely primarily on accuracy as the primary performance metric, while providing limited insight into how participants allocate time and cognitive effort during problem solving. Prior research has shown that participants from different cultural contexts may differ in their response time and accuracy patterns when completing non-verbal reasoning tasks (Ackerman et al., 2023). The present study therefore examines the newly developed MaRs-IB, a matrix reasoning task that incorporates both response time and accuracy, as a potentially valuable tool for examining cross-cultural variation in reasoning performance and task engagement in a more process-sensitive manner.

### 1.1. Cultural Frameworks of Intelligence, Task Engagement, and Response Timing: An East–West Perspective

Traditional psychological models have tended to conceptualize intelligence as a universal, stable, and decontextualized trait (Sternberg, 2021; Holden & Tanenbaum, 2023), thereby often interpreting cross-cultural differences in IQ or nonverbal reasoning performance primarily in terms of genetic and environmental influences (Rindermann et al., 2016). This perspective not only emphasizes a narrow set of influences on intelligence but also overlooks the substantial role of culture in shaping how intelligence is defined, valued, assessed, and expressed (Sternberg, 2004). Indeed, a growing body of work argues that intelligence is culturally bound, with cultural norms strongly influencing what is considered intelligent behavior (Earley & Ang, 2003).

Empirical research has documented systematic cross-cultural variation in conceptions of intelligence (Wilson & Mujtaba, 2008), with the East–West distinction being particularly prominent. In Confucian-influenced Eastern cultures, intelligence is often associated with diligence, moral integrity, and humility (Sternberg, 2003), whereas Western societies tend to emphasize verbal ability, processing speed, and learning capacity (Wilson & Mujtaba, 2008). Non-Western samples, including African and Asian populations, frequently highlight social competence and harmonious interpersonal functioning as central components of intelligence (Ruzgis & Grigorenko, 1994). For example, Korean participants describe intelligence using significantly more socially oriented traits than American participants (Lim et al., 2002).

These cultural differences have implications for standardized assessments of intelligence and abstract reasoning. Matrix reasoning tests, which primarily target cognitive–analytic components of intelligence, may provide an incomplete characterization of intelligence in cultural contexts where social, motivational, and personality-related attributes are viewed as integral. This issue may be particularly relevant in some East Asian cultural contexts, where modesty norms and reduced emphasis on self-promotion may shape how individuals evaluate and express their abilities (Yamagishi et al., 2012). Consistent with this, Furnham et al. (2012) found that mainland Chinese undergraduates rated themselves lower than UK undergraduates across multiple domains of intelligence, including linguistic, mathematical, creative, and nonverbal reasoning abilities.

Beyond differences in conceptualization, cultural frameworks may also shape how intelligence is enacted during testing. That is, individuals from different cultural backgrounds may differ in how they engage with standardized cognitive tasks, including variation in attentional focus, task engagement, and the allocation of cognitive effort during problem solving. For example, Šašinková et al. (2023) examined oculomotor patterns during object processing in complex visual environments and found that participants of Eastern descent (Taiwan) exhibited a more holistic processing style compared to Western participants (Western Turkey), who showed a more analytic processing style. In another study, Kelkar et al. (2013) contrasted Eastern (Indian-born) and Western (American-born) adults on standardized executive functioning and reasoning tasks and showed that Western participants showed greater reliance on analytic, rule-based processing in some verbal reasoning tasks, whereas Eastern participants demonstrated relatively stronger performance on perceptual trail-making tasks, consistent with differences in attentional orientation. Kuwabara and Smith (2012) further showed that Eastern and Western participants may exhibit different attentional and problem-solving tendencies early in development, with Japanese children exhibiting a relational attentional style that facilitated performance on structure-dependent tasks, and U.S. children demonstrating a relatively more object-focused attentional orientation that conferred advantages in object search tasks, highlighting the possibility that culturally shaped approaches to cognitive problem solving may contribute to observed performance differences.

Taken together, these findings suggest that relying solely on final performance scores, which is typical of many abstract reasoning measures, may obscure meaningful cross-cultural differences in approach, effort allocation, and decision-making during task performance. Assessing process-level variation in how participants engage with reasoning tasks may therefore provide a more informative basis for understanding cross-cultural variation in abstract reasoning.

In addition, intelligence frameworks, such as the Carroll-Horn model, recognize processing speed (often operationalized as reaction time or decision latency) as an important component of intelligence (Carroll, 1997; Papadopoulos et al., 2018). Processing speed is often conceptualized as reflecting the efficiency of information processing, and several studies have shown it to be correlated with general cognitive abilities (Schubert et al., 2022; Sheppard & Vernon, 2008), although response timing may also capture persistence, deliberative processing, and effort allocation depending on task structure and testing context (Alós-Ferrer & Buckenmaier, 2021). Research has also documented cross-cultural variation in response timing and processing speed across cognitive tasks (Kail et al., 2013). These findings highlight the importance of considering response time alongside accuracy when interpreting cross-cultural differences in reasoning performance.

### 1.2. Cultural Influences on Matrix Reasoning Task Performance

Considering the above concerns regarding cross-cultural differences in conceptions of intelligence, approaches to problem-solving, and processing speed, it follows that cultural factors are likely to influence performance on widely used abstract reasoning assessments. Matrix reasoning tasks remain the most commonly used, widely accessible instruments to assess abstract reasoning to date (Laurence & Macedo, 2023). Popular matrix reasoning tasks include The Cattell Culture Fair Intelligence Test (Cattell, 1940), the Raven’s Progressive Matrices (Raven, 2000; Raven et al., 2000/2024) and the matrix reasoning subtests embedded in the Wechsler batteries (Raven, 2000; Wechsler, 2011). These tasks typically present individuals with a series of visual patterns arranged in a grid, with one element missing. Individuals are then asked to identify the underlying rule or relational structure governing the patterns and select the correct option that completes the matrix. In general, matrix reasoning tasks have been endorsed as a preferred mode of assessment as compared to verbal tasks, as they are assumed to provide a more culturally fair measure of fluid intelligence, given that they are non-verbal and the stimuli used are typically limited to elementary shapes (Gonthier, 2022).

However, the long-standing assumption that matrix reasoning tests are culturally fair has been increasingly challenged. Early work documented systematic performance differences in abstract reasoning across ethnic and cultural groups, with some groups consistently outperforming others (Lynn & Vanhanen, 2002; Rushton & Jensen, 2005). Historically, such differences were often debated in relation to both genetic and environmental explanations of intelligence (Herrnstein & Murray, 1994), although broader consensus statements in the field have cautioned against attributing between-group differences to genetic factors alone (Neisser et al., 1996; Gottfredson, 1997). More recent research instead suggests that the matrix reasoning tasks used to assess intelligence in these populations may not be as culturally neutral as previously assumed. Rather than reflecting inherent cognitive differences, observed group disparities may arise from cultural factors embedded in test content, format, and response demands (van de Vijver & Tanzer, 2004; Gonthier, 2022). For example, studies involving African samples have shown that although participants often perform less well than Western samples on visuospatial matrix reasoning tasks, these differences are not consistently observed across other cognitive domains, including verbal measures of intelligence, suggesting that poorer performance on matrix reasoning tasks may reflect cultural familiarity with task formats rather than lower reasoning ability per se (Gonthier, 2022; Shuttleworth-Edwards et al., 2004).

These findings suggest that performance on matrix reasoning tasks may reflect not only underlying reasoning ability, but also variation in task engagement, persistence, response timing, and the regulation of cognitive effort during problem solving (Heine et al., 2001; Sternberg, 2004; Kitayama et al., 2009). Prior cross-cultural research has suggested that individuals from different cultural and educational contexts may differ in how they approach cognitively demanding tasks, including the extent to which they sustain effort, tolerate uncertainty, or invest additional time on difficult items (Kitayama et al., 2009). Such differences may contribute to observed variation in response timing and accuracy patterns on matrix reasoning tasks.

However, most existing matrix reasoning measures rely primarily on accuracy scores and therefore provide limited insight into process-level variation in how participants engage with reasoning tasks (Chierchia et al., 2019; Zorowitz et al., 2024). As Goldhammer and Entink (2011) demonstrated, reasoning speed and reasoning accuracy reflect partially distinct aspects of performance, with individuals often differing substantially in response timing despite comparable levels of accuracy, suggesting that response time may capture meaningful variation in engagement and deliberative processing during reasoning tasks. Incorporating response time, therefore, provides additional information about persistence, deliberative processing, and effort allocation during reasoning tasks, offering a more sensitive framework for examining cross-cultural variation in reasoning performance.

### 1.3. Testing the MaRs-IB Across Cultural Contexts

The MaRs-IB was originally developed for use in an online training study by Knoll et al. (2016) but has since been used as an abstract reasoning measure in other studies that demonstrate strong psychometric feasibility and reliability (Chierchia et al., 2019; Zorowitz et al., 2024). One advantage of the MaRs-IB is that it allows for the simultaneous examination of two dimensions of performance: accuracy and response time. This multidimensional approach provides a more process-sensitive perspective on reasoning performance by enabling researchers to examine how individuals allocate time and cognitive effort during problem solving. Prior work using the MaRs-IB has shown that higher accuracy is often associated with longer response times on more difficult items, suggesting that persistence and sustained cognitive engagement may contribute importantly to successful performance (Zorowitz et al., 2024).

Further, the MaRs-IB reduces practice effects from repeated exposure by drawing on a large item bank of 80 matrix reasoning puzzles that can be administered in multiple subset configurations. Meta-analytic evidence shows that repeated administrations of cognitive ability tests are associated with systematic score increases attributable to retest or practice effects rather than true changes in ability (Scharfen et al., 2018). The MaRs-IB mitigates this concern by allowing experimenters to administer different, non-overlapping puzzle subsets at pre- and post-test, thereby substantially reducing test–retest practice effects.

In their initial validation of the MaRs-IB, Chierchia et al. (2019) examined the psychometric properties of the task in a sample of 659 adults, adolescents, and children who were given eight minutes to complete as many items as possible. All participants received the items in a fixed order. The study reported good split-half and test–retest reliability, and MaRs-IB performance showed moderate correlations with both a working-memory task and the International Cognitive Ability Resource (ICAR) matrix-reasoning test, demonstrating satisfactory convergent validity. To facilitate further research, the authors publicly released summary statistics of item difficulty (i.e., proportion correct) for every template and clone, allowing other researchers to construct customized tests of varying difficulty and duration. Importantly, unlike the original validation study by Chierchia et al. (2019), in which participants completed as many items as possible within a fixed time limit, the present study administered all 80 items without a global time constraint. Accordingly, the present design did not explicitly impose a classical speed–accuracy trade-off and instead provided greater opportunity to examine variation in response timing, persistence, and effort allocation during reasoning performance.

Zorowitz et al. (2024) conducted another larger-scale assessment of the MaRs-IB using item response theory (IRT) with 1501 participants. Applying additive multilevel item structure models, they confirmed that the MaRs-IB exhibits several desirable psychometric characteristics: its items span a broad range of difficulty levels, demonstrate medium-to-large discrimination power, and show strong associations between item complexity and difficulty. The study also revealed that item clones are not always psychometrically equivalent and thus might not be assumed to be interchangeable. Notably, Zorowitz et al. (2024) observed that higher accuracy was associated with longer response times, particularly on more difficult items. This pattern suggests that persistence and willingness to invest cognitive effort may contribute importantly to successful reasoning performance, supporting the view that response time may index sustained engagement and deliberative processing rather than merely inefficient responding.

## 2. The Present Study

As illustrated above, prior work in English-speaking samples has provided strong validation evidence for the MaRs-IB and established it as a promising open-access item bank (Chierchia et al., 2019; Zorowitz et al., 2024). However, despite its growing use internationally as a measure of intelligence (Moses-Payne et al., 2022; Lowes et al., 2024), its measurement equivalence across cultural contexts remains untested. Given well-documented East–West differences in how intelligence is conceptualized and expressed, particularly via response time-accuracy patterns (Kelkar et al., 2013; Kuwabara & Smith, 2012; Kail et al., 2013), an East–West comparison provides a useful context for examining whether the MaRs-IB captures abstract reasoning in a culturally comparable way. Notably, its joint incorporation of accuracy and response time may help capture cross-cultural variation in how participants allocate time and engage with reasoning tasks.

As such, the present study tests the MaRs-IB in an East–West comparison. Participants from the UK and China were recruited as part of a larger study and completed the MaRs-IB online. We examined whether jointly modeling response time and accuracy supports a more process-sensitive framework for examining abstract reasoning across cultures, given evidence that individuals from different cultural contexts may differ in persistence, task engagement, and the allocation of cognitive effort during problem solving. More broadly, the MaRs-IB provides a useful framework for examining how participants from different cultural contexts may differ in their approaches to task engagement, persistence, and time allocation during reasoning tasks.

We first use Item Response Theory (IRT) to evaluate measurement equivalence across the two groups. We then examine the relationship between response time and accuracy to investigate potential cross-cultural differences in task engagement, response pacing, and effort allocation during reasoning performance. This study addresses the following research questions:

### 2.1. Key Research Questions

1. Does the MaRs-IB task demonstrate comparable difficulty and reliability in China and the UK?
2. Are performance differences between participants from China and the UK associated with differences in response timing, task engagement, and allocation of cognitive effort during reasoning performance?

### 2.2. Method and Participants

We analyzed cross-cultural assessment data from a larger cognitive study (for pre-registration details, see: https://osf.io/db8fz/ (accessed on 20 January 2026). Participants were recruited online from the UK and China from the general population. We excluded participants with an average response time below 2.5 s^1^, resulting in a final sample of 458 out of 493 participants included in the current analysis. The UK participants’ (*n* = 235; female = 116, male = 112, unidentified = 7), *M_age_* = 23.65 years (*SD* = 6.79). The Chinese participants’ (*n* = 222; female = 98, male = 106, unidentified = 19) *M_age_* = 22.90 years (*SD* = 3.55).

In terms of educational background, we attempted to recruit participants with roughly comparable educational profiles across the two countries. The UK sample predominantly consisted of participants holding a Bachelor’s degree (*n* = 145), followed by those with Master’s degrees (*n* = 47), Doctoral degrees (*n* = 24), and PGCE qualifications (*n* = 4). A smaller number of participants reported A-Level qualifications (*n* = 11), GCSE/O-Levels (*n* = 5), or partial doctoral-level study (*n* = 1). Similarly, the Chinese sample primarily comprised participants with Bachelor’s degrees (*n* = 106), Master’s degrees (*n* = 43), and Doctoral degrees (*n* = 45). One participant reported holding an associate degree, while 40 participants did not report their educational background.

This study was conducted in accordance with the ethical guidelines of Institutional Review Board of Ethics Committee of the Faculty of Education, University of Cambridge and was approved by the University’s Ethics Committee in July 2021 (protocol code CAMEDE-0221-96900, approved on 25 February 2021). All participants provided informed consent before the study commenced, and only healthy participants were recruited. The study was conducted in full compliance with the ethical standards of the British Psychological Society and the British Educational Research Association.

### 2.3. MaRs-IB Task Design

Following the design of Chierchia et al. (2019), each MaRs-IB item consisted of a 3 × 3 matrix featuring abstract shapes in eight cells and a blank bottom-right cell. Participants were required to infer the underlying rules governing four dimensions: shape, color, size, and position, and select the correct missing piece from four available options. Each trial followed a fixed sequence consisting of a 500 ms fixation cross, a 100 ms blank screen, and a maximum of 30 s to solve the matrix problem. To facilitate time management, a countdown timer appeared when only 5 s remained. The trial concluded either when a response was made or when the time limit was reached, followed by participant feedback. Unlike the original MaRs-IB design, which imposed a fixed global time limit and presented all items in a fixed sequential order, our study randomly presented all 80 items. This modification was implemented to improve internal validity and reduce potential systematic bias associated with sequential item presentation and order effects (Shadish et al., 2002; Atmanspacher & Römer, 2012). In addition, this procedure ensured that participants from both countries completed the same set of items. Because all participants completed the same fixed set of items, total scores directly reflected overall accuracy. Furthermore, response time for each individual item was also recorded.

### 2.4. Procedure

The testing procedure has been described in detail elsewhere (see OSF: https://osf.io/fy8aw/ (accessed on 20 January 2026) as part of a large-scale study on cognitive function and scientific ability comprising nine computer-based tasks. The total duration of the assessment was approximately 70 min. To maintain consistency across participants, the tasks were administered in a fixed sequence, with the MaRs-IB consistently placed as the fifth task in the battery. The MaRs-IB was developed by the research team using materials adapted from Chierchia et al. (2019) and was programmed and administered online via PsychoPy. Participants received standardized verbal instructions at the start of the session before completing the MaRs-IB.

## 3. Data Analysis

All data processing and analyses were conducted in the R (R Core Team, 2025) environment. Data were cleaned using the *tidyverse* package (Wickham et al., 2019). We then computed descriptive statistics to summarize performance patterns and evaluated item-level reliability using the *psych* (Revelle, 2025) and *performance* packages (Lüdecke et al., 2021a).

To examine the cross-cultural comparability of the MaRs-IB in China and the UK, we analyzed item responses using Item Response Theory (IRT). IRT enables estimation of participants’ latent ability and tests whether items function similarly across groups, thereby evaluating measurement equivalence. Prior to model estimation, the data were examined to ensure that key IRT assumptions of unidimensionality and local independence were met prior to model estimation (Embretson & Reise, 2000; De Ayala, 2021). Model comparisons supported a one-factor structure, and Yen’s Q3 statistics indicated no substantial local dependence (Yen, 1984), thus deeming the data suitable for IRT analysis. As responses were dichotomous (0–1 scale), standard logistic IRT models were fitted to the data, including the two-parameter (2PL) and three-parameter (3PL) models. Model selection was based on the Akaike’s Information Criterion (AIC; Akaike, 1974) and Bayesian Information Criterion (BIC; Schwarz, 1978) having the lowest values, and item-level fit was assessed using the S-Chi-square statistic (Orlando & Thissen, 2003), applying a significance level of *p* < .01 (Flens et al., 2017). All IRT analyses were conducted in the *mirt* package (Chalmers, 2012).

Next, using the selected IRT model, we conducted a Differential Item Functioning (DIF) analysis. Lord’s chi-square test (Cohen & Kim, 1993; Lord, 1980) evaluated whether item responses differed by country, using *p* < .01 (Xi et al., 2020). Items showing significant DIF were removed, and reliability was re-estimated on the retained item set. Following this, and considering that our sample size was relatively small, we estimated the latent ability scores of all participants using the Expected a Posteriori method implemented in the *mirt* package (Chalmers, 2012) and plotted the distribution of the estimated abilities.

Finally, referencing Zorowitz et al.’s (2024) procedure, we performed a linear mixed-effects model (LMM) using the *lme4* package (Bates et al., 2015) to examine how response time was predicted by response accuracy, overall performance level (rest score), item difficulty, and country to examine how response time varied as a function of response accuracy, item difficulty, overall performance level, and country.

To ensure the statistical validity of using LMM, we performed several assumption checks using the *performance* (Lüdecke et al., 2021a) and *see* (Lüdecke et al., 2021b) packages. First, the linearity assumption was verified via residual-versus-fitted plots to ensure that the relationship between predictors and the outcome was appropriately captured by the linear functional form. Second, considering the skewed data on response time, following Zorowitz et al. (2024), we conducted a log transformation of the response time data to mitigate the inherent right-skewness issue. Third, the homoscedasticity (homogeneity of variance) of residuals was assessed to confirm that the error variance remained stable across the range of predicted values. Visual inspections of the diagnostic plots indicated that these assumptions were well satisfied (see Supplementary Materials Section S1).

Consequently, the log-transformed LMM was deemed robust and appropriate for the subsequent hypothesis testing.

In the LMM, to account for potential demographic influences, participants’ age, gender and country were included as control variables; we then specified the fixed-effects structure to include higher-order interactions between country, accuracy, rest score and item difficulty. Regarding the random-effects structure, we specified random intercepts for participants (1|ParticipantID) following Zorowitz et al. (2024). While some frameworks suggest including random intercepts for items, we determined that our current structure was parsimonious and sufficient to account for the non-independence of observations nested within individuals, thereby controlling for idiosyncratic variation in baseline response speeds (Baayen et al., 2008). Model parameters were estimated using Restricted Maximum Likelihood, which provides unbiased estimates of variance components (Bates et al., 2015).

Data and analysis scripts are available on the Open Science Framework (see: https://osf.io/db8fz/ (accessed on 20 January 2026)).

## 4. Results

### 4.1. Descriptive Statistics

We first report descriptive statistics for accuracy, response time, and inverse efficiency score for all participants (see Table 1 for details). Participants from China demonstrated higher mean accuracy, represented as proportions correct (*M_accuracy_* = 0.72, *SD_accuracy_* = 0.17) in comparison to participants from the UK (*M_accuracy_* = 0.57, *SD_accuracy_* = 0.18). In terms of absolute performance metrics, this pattern corresponds to an average of 57.81 correctly solved items (*SD* = 13.88) for the Chinese sample and 45.76 items (*SD* = 14.03) for the UK sample. It is worth noting that because all participants across both cohorts fully completed the entire 80-item assessment without omission, the absolute number of correct items represents a direct linear transformation of the overall accuracy scores (Correct Items = 80 × Accuracy). Accordingly, the observed between-group difference in correctly solved items reflects the same underlying performance pattern captured by the accuracy analysis.

Chinese participants also had significantly longer response times for correct trials (*M_RT_* = 11.40 s, *SD_RT_* = 2.89) than UK participants (*M_RT_* = 8.56 s, *SD_RT_* = 3.26). Notably, the mean response time in the UK sample was comparable to previously reported latencies for correct trials (e.g., *M_RT_* = 7.91 s), yet overall accuracy diverged from the 69.15% correct rate reported for the UK adult sample in Chierchia et al. (2019). This discrepancy may reflect differences in data filtering: we excluded participants with mean response times below 2.5 s, whereas prior work may have applied trial-level exclusions. The inverse efficiency score, which combines accuracy and response time, was slightly higher for Chinese participants (*M_efficiency_* = 16.19, *SD_efficiency_* = 3.99) than for UK participants (*M_efficiency_* = 15.27, *SD_efficiency_* = 5.04). Correlation analyses revealed significant relationships between accuracy and response time across both China (r = 0.60, *p* < .001) and the UK (r = 0.61, *p* < .001).

**Figure 1:**
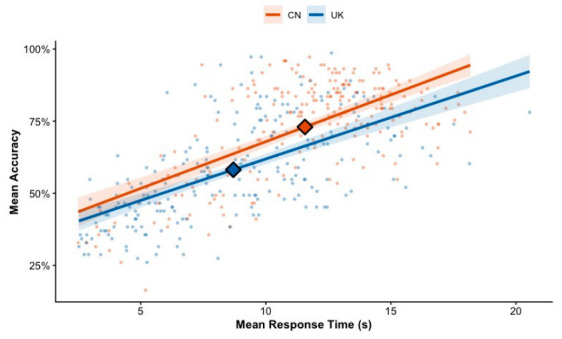
Participant-level distribution of mean accuracy (Y) and response time (X) by country. Notes. Individual data points represent participant-level means, plotted with Mean Response Time (s) on the X-axis and Mean Accuracy on the Y-axis. Orange markers and lines denote the Chinese (CN) sample, while blue markers and lines denote the UK sample. Solid lines represent linear regression fits for each group, with shaded areas indicating 95% confidence intervals. The large, bordered diamond symbols represent the respective group centroids (joint group means), illustrating the clear segregation between the two cohorts.

**Figure 2:**
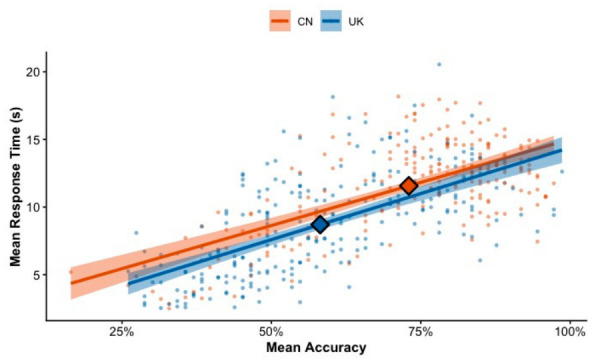
Participant-level distribution of mean accuracy (X) and response time (Y) by country. Notes. Individual data points represent participant-level means, plotted in the symmetrical representation with Mean Accuracy on the X-axis and Mean Response Time(s) on the Y-axis. Orange markers and lines denote the Chinese (CN) sample, while blue markers and lines denote the UK sample. Solid lines represent linear regression fits for each group, with shaded areas indicating 95% confidence intervals. The large, bordered diamond symbols represent the respective group centroids (joint group means), illustrating the clear segregation between the two cohorts.

To further visualize these cognitive profiles, we plotted participant-level mean accuracy against mean response time by country to examine the relationship between response time and accuracy across the two groups, with response time on the X-axis and accuracy on the Y-axis (Figure 1). As illustrated, the regression lines for both groups exhibit a positive slope, linking longer processing time to higher accuracy. Crucially, however, the two regression lines do not overlap to form a single continuous response time-accuracy function. Instead, they exhibit a distinct vertical displacement: at any given level of response time, participants from the Chinese sample demonstrate systematically higher accuracy than their UK counterparts. This parallel separation—further accentuated by the clear segregation of the group centroids—suggests that the cross-national difference is not a simple strategic trade-off along a shared performance curve. Instead, it points to systematic between-group differences in response timing, task engagement, persistence, or decision thresholds. To ensure analytical symmetry and comprehensively evaluate these cognitive profiles, we reversed the axis configuration to project Mean Accuracy on the X-axis and Mean Response Time on the Y-axis (Figure 2). This inverse representation allows us to examine how response timing varied across comparable levels of performance accuracy in the two groups. As illustrated, the regression line for the Chinese cohort (CN) exhibits a systematic upward vertical displacement relative to the UK cohort across the entire accuracy spectrum.

Crucially, at comparable levels of performance accuracy (e.g., at the 75% correct benchmark), participants from the Chinese sample allocate longer response times than their UK counterparts systematically. Together with the patterns observed in Figure 1, these findings suggest systematic between-group differences in response timing and task engagement during reasoning performance. In particular, the Chinese sample appeared more willing to continue investing time on difficult items, whereas the UK sample demonstrated relatively faster responding across performance levels.

### 4.2. Reliability

Split-half reliability was high in both samples (China: 0.93; UK: 0.92 after Spearman–Brown correction). Seven items were flagged for DIF and were removed from subsequent analyses (see below). Spearman–Brown-corrected split-half reliability remained high (China: 0.92; UK: 0.93) despite item removal. Overall, these indices indicate that the MaRs-IB maintains strong internal consistency across cultures, both prior to and after excluding items showing differential functioning.

### 4.3. Item Response Theory Analysis

#### Model Fit and Item Fit

We compared the fit of the 2PL and 3PL models (Table 2). The results indicate that the 2PL model provided a generally better model fit for both the UK and China datasets. Specifically, in the UK sample, while AIC favored the 3PL model, BIC favored the more parsimonious 2PL model. BIC is often preferred in model selection as it provides a more stringent penalty for model complexity than AIC, helping to avoid overfitting (Schwarz, 1978; Vrieze, 2012). Furthermore, considering that stable estimation of 3PL parameters (e.g., the guessing parameter) typically requires larger sample sizes than those currently available in our study to ensure accuracy and interpretability (De Ayala, 2021), we finally selected the 2PL model for all subsequent analyses to ensure both model stability and cross-sample consistency. We then evaluated item-level fit under the 2PL model; all items demonstrated acceptable fit, with *chi-square* tests non-significant at *p* > 0.01. Detailed item-fit results are provided in Supplementary Materials Section S2.

### 4.4. Differential Item Functioning Analysis

To examine item-level measurement invariance across countries, Lord’s chi-square statistic was computed for each of the 80 items. Seven items showed significant country differences (*p* < .01), indicating potential differential item functioning: item 6 (*χ*^2^ = 12.28, *p* = .002), item 17 (*χ*^2^ = 9.43, *p* = .009), item 18 (*χ*^2^ = 9.95, *p* = .007), item 61 (*χ*^2^ = 11.19, *p* = .004), item 66 (*χ*^2^ = 10.75, *p* = .005), item 69 (*χ*^2^ = 9.53, *p* = .009), and item 77 (*χ*^2^ = 11.41, *p* = .003). These items were removed prior to cross-national comparisons to strengthen the validity of subsequent analyses, resulting in 73 invariant items for further analyses. Full DIF results are reported in Supplementary Materials Section S3.

### 4.5. Item Parameter Analysis

Based on the IRT analysis (Table 3), most items showed moderate-to-high discrimination (a ≥ 0.65): 83.56% in the UK and 94.53% in China, indicating that the majority of items effectively differentiate participants across ability levels. For difficulty (*b*), defined as the level (*theta*) associated with a 50% probability of a correct response, 76.71% of UK items and 42.47% of China items were classified as more than moderately difficult. This pattern suggests that the item set was, on average, easier for Chinese participants than for UK participants. Overall, the IRT analysis supports good item quality in both samples, although a small subset of items exhibited cross-country inconsistencies in discrimination.

### 4.6. Ability Distribution

**Figure 3:**
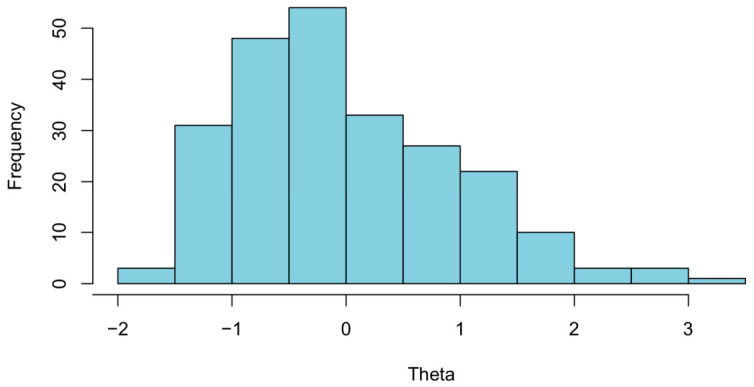
Ability Distribution Histogram for UK Participants. Note. Theta = ability estimate; higher *theta* indicates higher ability on the latent trait being measured; Frequency = Number of Participants.

**Figure 4:**
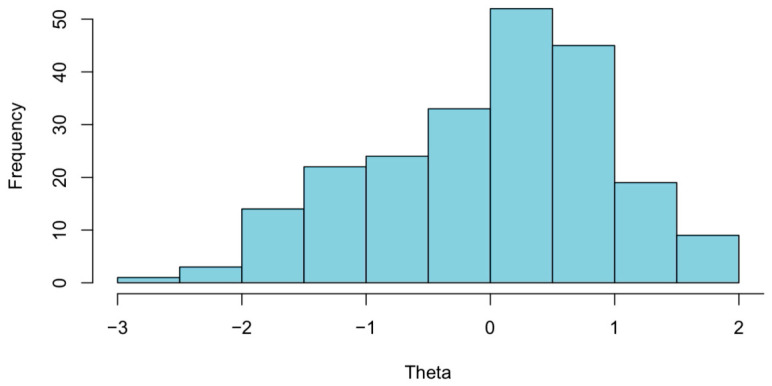
Ability Distribution Histogram for Chinese Participants. Note. Theta = ability estimate; higher *theta* indicates higher ability on the latent trait being measured; Frequency = Number of Participants.

Following the DIF analysis, we estimated participants’ latent ability (*theta*) scores in both countries (Figure 3 and Figure 4). The resulting histograms revealed that 70.64% of UK participants had *theta* values between −1.5 and 0.5, whereas 68.94% of Chinese participants’ *theta* values fell between −1.0 and 1.0. Overall, the Chinese showed a more centrally clustered ability distribution, while the UK distribution was left-skewed, with a greater concentration of scores at the lower end of the *theta* scale.

**Figure 5:**
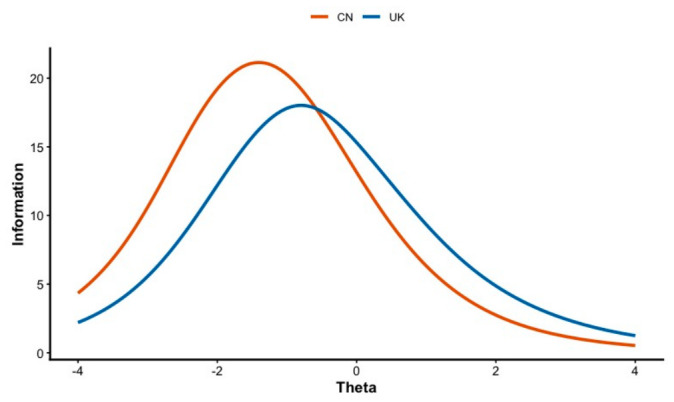
Test Information Curves (TIC) for Chinese and UK samples following Differential Item Functioning (DIF) adjustment. Note. Theta = ability estimate; higher theta indicates higher ability on the latent trait being measured; Information = the measurement precision of the test; higher Information values indicate lower standard errors of measurement and greater reliability at that specific ability level. The orange curve represents the Chinese sample (CN), and the blue curve represents the UK sample.

To further evaluate whether the instrument maintained comparable precision across the different ability ranges where the two samples were concentrated, we examined the Test Information Curves (TIC) post-DIF adjustment (see Figure 5).

The TIC illustrates that for both groups, the MaRs-IB provides maximum measurement information within the theta range of −2.0 to 1.0. Critically, this high-precision interval directly overlaps with the areas of highest participant density identified in our distribution analysis (i.e., the −1.5 to 0.5 range for the UK and −1.0 to 1.0 for China). Although the Chinese sample exhibited a higher peak in information, the overlapping curves demonstrate that the instrument is sufficiently and comparably precise across the specific theta ranges where both UK and Chinese participants are primarily concentrated. This ensures that the observed cross-cultural differences in ability distributions are not artifacts of measurement error but reflect robust differences in latent performance.

### 4.7. Linear Mixed-Effects Model of Response Times

The results of the linear mixed-effects model predicting response time are summarized in Table 4.

After controlling for the main effects of age (*β* = 0.008, *t* = 2.151, *p* = .031) and gender (*β* = 0.012, *t* = 0.305, *p* = .760) and country (*β* = 0.658, *t* = 2.882, *p* = .004). We observed that response times were significantly longer for correct responses (*β* = 1.427, *t* = 10.067, *p* < .001) and for participants with higher rest scores (*β* = 0.035, *t* = 10.181, *p* < .001). These results indicate that participants generally allocated more time to trials they answered correctly and that overall higher-performing individuals spent more time processing items. However, the main effect of item difficulty was not significant (*β* = 0.338, *t* = 1.050, *p* = .294), indicating that the impact of item difficulty was not uniform across the entire sample.

In terms of interaction results, we found several cross-country differences in observed performance with China as the reference group. We found a significant interaction between country × accuracy (*β* = −0.613, *t* = −3.170, *p* = .002), showing that relative to Chinese participants, UK participants exhibited a more pronounced speed-up on correct trials. Conversely, Chinese participants maintained a more sustained and cautious temporal investment even when answering correctly, consistent with the possibility that Chinese participants maintained greater persistence or sustained engagement during reasoning performance. In addition, the significant interaction between country × rest score (*β* = −0.014, *t* = −2.741, *p* = .006) suggested that higher-performing UK participants tended to respond relatively faster than higher-performing Chinese participants.

We observed a significant interaction effect between accuracy and rest score, indicating that the difference in response time between correct and incorrect trials varied according to participants’ general task proficiency. The rest score and item difficulty interaction was also significant (*β* = 0.031, *t* = 4.507, *p* < .001), showing that the effect of item difficulty on response time was more pronounced among participants with higher rest scores.

Finally, we found a significant 3-way interaction among accuracy, rest score, and item difficulty (*β* = 0.026, *t* = 2.973, *p* = .003), suggesting that the extent to which correct and incorrect responses differed in response time was not constant across tasks, but depended on how difficult the item was and how well participants generally performed on the task.

Overall, rather than supporting a traditional strategic speed–accuracy trade-off on a shared performance curve, these LMM results provide trial-level verification of the systemic, cross-national proficiency differences observed in our participant-level analysis (Figure 1). To formally test whether this cross-national divergence represents a flexible strategic shift along a common performance trajectory or a systematic group-level difference in processing efficiency, we executed a participant-level model comparison. Specifically, we contrasted a constrained baseline model assuming a single, shared response time-accuracy function (Accuracy ~ Speed) against a full interaction model allowing group-specific intercepts and slopes (Accuracy ~ Speed × Country). The formal model comparison via an incremental F-test demonstrated that the interaction model provided a significantly superior fit to the data than the response time-accuracy model (see Supplementary Materials Section S4 for complete regression coefficients and ANOVA tables). Overall, these findings suggest that the observed cross-cultural differences were not fully captured by a single shared response-time and accuracy function. Instead, the results indicate systematic between-group differences in how participants allocated time and engaged with reasoning tasks. Consistent with the broader findings of the present study, the Chinese sample appeared more willing to sustain effort and continue investing time on challenging items, whereas the UK sample demonstrated relatively faster responding across task conditions.

## 5. Discussion

In this study, we aimed to examine the performance of the MaRs-IB across adult samples from the UK and China, with a particular focus on its psychometric robustness and cross-group comparability, as well as to understand any potential differences in task engagement and response timing patterns across UK and China cultural groups. Results indicate that the MaRs-IB demonstrates strong psychometric properties and high reliability in both countries, supporting its suitability for cross-cultural use in adult populations. Differential item functioning analyses revealed limited evidence of item bias across groups, suggesting broad measurement equivalence; nevertheless, systematic group differences in overall task performance were observed. Specifically, cross-group comparisons on the MaRs-IB indicated differences in accuracy, response time, and their interaction, pointing to distinct response time and accuracy patterns in MaRs-IB performance between the UK and Chinese samples. In sum, our findings support earlier studies (Chierchia et al., 2019; Zorowitz et al., 2024) by demonstrating that the robustness of the MaRs-IB extends beyond single-country samples and holds across cultural boundaries in adult populations. However, subsequent results revealed notable differences in observed performance distributions between UK and Chinese participants, suggesting possible cross-cultural variation in the metacognitive regulation of effort, task engagement, and time allocation during reasoning. The following sections elaborate on the robustness of the MaRs-IB and its potential applications in mainstream research and then consider possible interpretations of the observed differences between UK and Chinese participants. Importantly, although the observed performance patterns are interpreted in relation to prior cross-cultural literature on cognition, motivation, and educational socialization, the present study did not directly measure these constructs. Accordingly, the interpretations discussed below should be understood as theoretically informed hypotheses that are broadly consistent with the observed data rather than as direct explanations of the mechanisms underlying group differences.

### 5.1. Robustness and Research Applications of the MaRs-IB

Beyond questions of cross-group comparability, the present results support the psychometric robustness of the MaRs-IB when evaluated against criteria commonly applied to established matrix reasoning measures (e.g., Raven’s Progressive Matrices; White et al., 2022). Consistent with these widely used instruments, the MaRs-IB demonstrates strong internal reliability, largely stable item functioning, and a coherent latent structure across samples. Our results also align with Zorowitz et al. (2024), who reported well-calibrated item difficulty and discrimination, suggesting that the MaRs-IB can differentiate individuals across a broad range of reasoning abilities.

Importantly, the MaRs-IB extends traditional matrix reasoning tasks by incorporating process-sensitive indicators, including response time alongside accuracy, which enables fine-grained analysis of response timing, task engagement, and reasoning processes. This feature is particularly valuable for contemporary cognitive and developmental research that seeks to model not only reasoning outcomes but also reasoning processes (Klein Entink et al., 2009; Niebaum & Munakata, 2023). Coupled with item-level calibration and cross-group stability, these properties make the MaRs-IB well suited to multi-sample and cross-cultural research designs. Taken together, the MaRs-IB offers a psychometrically sound and methodologically modern complement to established abstract reasoning measures in mainstream research contexts.

### 5.2. Cross-Cultural Differences in MaRs-IB Performance Patterns

Our results suggest that the observed cross-cultural differences in MaRs-IB performance may reflect differences in how participants allocated time and cognitive effort during problem solving. Although both groups showed positive associations between response time and accuracy, Chinese participants were generally slower but more accurate, whereas UK participants responded more quickly but with lower overall accuracy. Rather than reflecting a classical speed–accuracy trade-off, these findings may instead indicate differences in persistence, deliberative responding, or metacognitive regulation of effort during reasoning tasks. These patterns may reflect cultural differences in beliefs about effort and ability that shape approaches to task engagement (Ackerman et al., 2023). For example, Wang et al. (2026) reported that Western (Canadian) participants tended to view both ability and effort as equally important in determining outcomes, whereas Eastern (Chinese) participants placed greater weight on effort. Furthermore, with age, Chinese participants increasingly attributed performance variation to effort, while Canadians continued to attribute outcomes to a combination of ability and effort. Such beliefs could potentially encourage greater time investment and sustained engagement on challenging items in contexts emphasizing effort, whereas contexts placing relatively greater emphasis on efficiency or stable ability may encourage faster responding.

Further, our results reveal notable differences in performance distributions between UK and Chinese participants. Specifically, Chinese participants exhibit a more centralized theta distribution, suggesting a more centrally clustered performance distribution. In contrast, UK participants show a left-skewed distribution, with a higher concentration of scores on the lower end of the scale. One possible interpretation is that the greater persistence observed among Chinese participants on challenging items in the MaRs-IB may be consistent with prior work suggesting stronger cultural emphasis on effort and sustained engagement over innate ability in some East Asian educational contexts (Wang et al., 2026). These findings indicate substantial between-group differences in observed performance; however, the present results also suggest that differences in persistence, effort allocation, and task engagement may contribute importantly to these performance patterns. Importantly, these differences were also reflected in the absolute number of correctly solved items. Chinese participants solved, on average, approximately 12 more items correctly than UK participants across the full 80-item assessment, corresponding to a substantial between-group effect. This indicates that the observed differences were not limited to subtle variation in response timing but extended to meaningful differences in overall task performance. At the same time, because all participants completed the full assessment without omission or strict global time constraints, the observed response-time patterns provide additional insight into how participants engaged with difficult items during reasoning performance. In this context, longer response times may reflect not only slower responding, but also greater persistence, sustained engagement, and continued cognitive effort on challenging items (Goldhammer & Entink, 2011; Zorowitz et al., 2024).

Research in Chinese educational and cultural contexts suggests that learners are more likely to endorse an incremental theory of intelligence, viewing ability as malleable and improvable through sustained effort (Chen & Wong, 2015; Dweck, 2000). In Confucian-influenced contexts, academic performance is often embedded in social expectations, family obligation, and moral self-development, which fosters both mastery-oriented learning and strong performance-approach goals (Chen & Wong, 2015; Salili & Lai, 2003). Such beliefs may encourage sustained effort across items, including more challenging ones, reducing premature disengagement and potentially contributing to a more centralized distribution of estimated ability.

Another possible interpretation draws on literature examining cross-cultural differences in cognitive style. Prior work has suggested that Eastern participants may, on average, adopt relatively more holistic and context-sensitive processing tendencies, which involve attending to relationships among elements and integrating multiple aspects of a problem before responding, whereas Western participants may more often favor analytic processing tendencies, which prioritize focal objects, rule-based reasoning, and selective attention to task-relevant features, often facilitating faster responses (Nisbett, 2003). Although these constructs were not directly measured in the present study, the observed response time–accuracy patterns are broadly consistent with the possibility that participants differed in how they engaged with the task, including how they allocated attention, sustained effort, and approached difficult items. Some prior research has proposed that certain East Asian educational settings may place relatively greater emphasis on accuracy, persistence, error avoidance, and careful verification of responses before committing to an answer (Varnum et al., 2010; Xu et al., 2021). Such norms may promote greater time investment on challenging items. In contrast, Western contexts more strongly emphasize analytic cognition, which involves focusing on discrete objects and rule-based reasoning, which may encourage greater focus on discrete task elements and relatively faster responding in some reasoning contexts (Nisbett et al., 2001). As a result, individuals from these cultural backgrounds may differ not only in observed performance levels, but also in how they allocate time and cognitive resources during task performance.

Another speculative possibility is that cultural differences in responses to errors and uncertainty could contribute to the greater persistence observed among Chinese participants on challenging items. Research on experiential avoidance and cultural values suggests that East Asian norms emphasizing self-discipline, contextual interdependence and sensitivity to social evaluation are associated with careful, deliberate processing and motivation to minimize unwanted outcomes such as errors (Yoon et al., 2024). If such norms were operative in the present context, they could potentially encourage participants to invest additional time verifying responses before committing to an answer, particularly on difficult items. Together, these findings suggest that East-Western differences in test performance may partly reflect culturally shaped attitudes toward error, effort, and task engagement.

One additional hypothesis is that differences in educational socialization may contribute to the observed performance patterns. Some work on exam-oriented educational systems has suggested that students may become accustomed to sustained effort and careful responding under evaluative conditions (Hau & Ho, 2010; Huang, 2025). In such contexts, students are socialized to approach assessments as opportunities requiring careful reasoning and continued engagement, even when task demands increase (Xu et al., 2021). This orientation may help explain why Chinese participants were more likely to work through difficult items rather than disengage under uncertainty. By contrast, UK participants’ faster response times and lower accuracy on more difficult items may reflect educational experiences that place greater emphasis on efficiency, rapid problem solving, and flexible problem-solving approaches. Consistent with this view, Western participants in cross-cultural executive functioning research completed complex cognitive tasks more rapidly than Eastern counterparts, a pattern attributed to analytic and rule-based tendencies associated with relatively faster responding (Kelkar et al., 2013; Zhang & Sternberg, 2005). In lower-stakes or unfamiliar testing contexts, Western participants may prioritize speed or intuitive responding over exhaustive deliberation, which can lead to earlier disengagement on cognitively demanding items (Murayama et al., 2013).

Overall, our results suggest that cross-cultural differences in matrix reasoning performance may reflect not only differences in observed performance levels, but also differences in how participants regulate effort, allocate time, and sustain engagement during cognitively demanding tasks, potentially influenced by educational and cultural contexts (Sun et al., 2021). Chinese participants appeared more willing to sustain effort and continue investing time on challenging items, whereas UK participants demonstrated relatively faster responding with lower overall accuracy. Importantly, although matrix reasoning tasks are often administered without strict time limits to enhance fairness, research shows that timing conditions substantially alter reasoning processes, task engagement patterns, and accuracy, even under mild time pressure (Gonthier, 2022). These findings suggest that performance on matrix tasks is sensitive to how individuals regulate their time and cognitive effort. Thus, removing strict time constraints may not fully eliminate cultural differences in self-regulated pacing and response timing patterns, which may continue to shape cross-cultural performance patterns. Future cross-cultural studies of matrix reasoning and fluid intelligence tasks should consider these patterns of task engagement and response timing when interpreting group differences.

## 6. Limitations and Future Directions

Several limitations of the present study suggest avenues for future research. First, the cross-sectional design constrains causal inferences about how educational and cultural contexts shape task engagement patterns; longitudinal work would help clarify developmental trajectories in response timing, persistence, and reasoning engagement over time. Second, although we examined psychometric properties extensively, we did not include direct measures of instructional practices, test familiarity, or specific educational histories, which limits precise explanations of why participants from different contexts adopt different patterns of task engagement. Future research incorporating detailed information about participants’ prior instruction, exposure to reasoning paradigms, and schooling practices would help to better explain the source of cross-cultural differences in task engagement and response timing. Third, as with many cross-cultural cognitive studies, unmeasured contextual variables such as language proficiency, test anxiety, or culturally shaped metacognitive styles may have contributed to performance patterns. For example, work on cultural influences on metacognition highlights that individuals from different societies can vary in their confidence, monitoring, and regulation of cognitive processes, which may in turn affect how they regulate cognitive effort and response timing under uncertainty (Ordin et al., 2024).

Methodologically, our findings also align with broader concerns in cross-cultural cognitive assessment literature that measurement invariance does not guarantee equivalence of cognitive processes. First, even tasks that show psychometric equivalence can be interpreted differently or engaged with differently depending on cultural and motivational contexts (Sternberg, 2004; Kusano et al., 2025). Second, the MaRs-IB implemented in this study was embedded within a relatively long test battery, which may have introduced motivation-related biases associated with task length or fatigue. Such effects could partially account for the fact that the average performance observed in the present study was lower than that reported in previous studies (Zorowitz et al., 2024).

Third, we notice that much of our reasoning regarding differences in performance patterns between the UK and Chinese groups is informed by previous literature suggesting potential cross-cultural variations grounded in cultural dimensions. These interpretations remain speculative, and future work could use process models to parse underlying decision parameters (Rao, 2010) and more precisely isolate the processes underlying participants’ task engagement and response timing in the MaRs-IB. Future studies could also examine how instructional and cultural contexts interact with motivational constructs such as implicit self-theories, achievement goals, and uncertainty avoidance, that prior work has linked to cultural differences in cognitive engagement and task persistence (Ordin et al., 2024; Noordzij et al., 2024; Bajpai, 2025).

Fourth, our sample consisted of a convenience sample collected entirely online. Although this approach was partly necessitated by the COVID-19 pandemic during the data collection period, such a sampling strategy may still introduce selection bias; for instance, online recruitment likely attracted individuals with higher levels of technological literacy (Andrade, 2021). Furthermore, reliance on this type of sample inevitably limits the generalizability of our findings beyond the specific demographic groups represented in the UK and Chinese samples (Salkind, 2010). Future research should employ more diverse or probabilistic sampling strategies to validate these cross-cultural findings across broader populations (Jager et al., 2017).

Fifth, our overall task battery was relatively long, and the MaRs-IB was administered in the middle of the session. Given that the MaRs-IB itself is a time-intensive task requiring substantial cognitive resources, its performance outcomes may have been influenced by cumulative cognitive load, which could lead to fatigue, attentional decline, or order effects. Future studies could employ counterbalanced designs or shorter assessment intervals to further mitigate these potential influences.

Overall, extending this research to other cognitive domains, age groups (including adolescents and older adults), and educational systems would clarify the generalizability of the observed patterns. Incorporating ethnographic or qualitative approaches could also illuminate how participants themselves interpret and approach test situations across cultural contexts. Taken together, while the present psychometric analyses strengthen confidence that the MaRs-IB functions similarly across groups, deeper investigations into contextual and process-level factors related to task engagement and effort regulation will be important for understanding how and why culturally linked differences in task engagement, persistence, and effort regulation emerge.

## 7. Conclusions

In summary, this study introduces the MaRs-IB, an online matrix reasoning instrument designed to assess abstract reasoning by jointly considering accuracy and response speed. Among participants from China and the UK, MaRs-IB demonstrated robust psychometric properties and high reliability, supporting its utility for cross-country research on fluid intelligence. Importantly, we observed systematic between-group differences in overall task performance. Rather than reflecting a classical speed–accuracy trade-off in which participants strategically sacrifice one dimension for another, the observed patterns may instead reflect cross-cultural differences in persistence, deliberative engagement, and the metacognitive regulation of cognitive effort during reasoning tasks. Specifically, Chinese participants appeared more willing to continue investing time on challenging items, whereas UK participants demonstrated relatively faster responding with lower overall accuracy.

These findings have two key implications for cross-cultural assessment. First, when cultural groups differ in patterns of task engagement, relying on accuracy as the sole performance criterion may yield biased interpretations, even for ostensibly “culture-neutral” figural tasks. Second, cross-cultural differences on matrix reasoning measures should be interpreted cautiously: they may reflect differences in persistence, task engagement, and the regulation of cognitive effort during problem solving rather than substantive differences in cognitive capacity. Future research should test the generalizability of these conclusions across additional cultural contexts, examine conditions that experimentally manipulate time constraints, and develop measurement models that explicitly distinguish latent reasoning performance from process-level differences in response timing, persistence, and effort allocation. In addition, future studies could incorporate targeted measures of cognitive processing styles, educational socialization, implicit theories of intelligence, motivational beliefs, and task engagement to better understand the psychological and cultural factors that may underlie cross-cultural differences in task engagement and response timing.

## Figures and Tables

**Table 1:** Descriptive Statistics (Raw Data).

Variables	UK	China
*n*	235	222
Age (mean)	23.65	22.93
Age (sd)	6.79	3.55
Age (min)	18	18
Age (max)	77	35
Accuracy (mean)	0.57	0.72
Accuracy (median)	0.55	0.78
Accuracy (sd)	0.18	0.17
Accuracy (min)	0.26	0.16
Accuracy (max)	0.98	0.96
Number of correct items (mean)	45.76	57.81
Number of correct items (sd)	14.03	13.88
Response Time ^1^ (mean)	8.56	11.40
Response Time (median)	8.70	11.87
Response Time (sd)	3.26	2.89
Response Time (min)	2.39	2.69
Response Time (max)	19.02	17.70
Efficiency (mean)	15.27	16.19
Efficiency (median)	14.22	15.52
Efficiency (sd)	5.04	3.99
Efficiency (min)	5.83	7.52
Efficiency (max)	34.37	30.68

Note. Response Time for correct trials, in seconds. ^1^ Response Time for correct trials in seconds.

**Table 2:** Model Fit Indices Comparison.

IRT Model	*AIC*	*BIC*
**UK**		
2PL	20,769.01	21,322.54
3PL	20,714.63	21,544.93
**China**		
2PL	15,430.79	15,934.39
3PL	15,515.04	16,270.44

Note. *AIC* = Akaike Information Criterion; *BIC* = Bayesian Information Criterion.

**Table 3:** Item Response Analysis After Item Removal.

UK	*a*	*b*	CN	*a*	*b*
item1	0.34	−6.37	item1	0.81	−2.96
item2	2.05	−1.61	item2	0.95	−2.94
item3	1.62	−2.17	item3	1.28	−3.40
item4	1.64	−1.57	item4	1.98	−2.27
item5	1.00	−2.71	item5	2.00	−2.27
item7	1.37	−2.01	item7	1.56	−2.63
item8	1.22	−1.78	item8	1.62	−2.06
item9	2.04	−0.86	item9	2.05	−1.43
item10	0.81	−0.72	item10	1.24	−1.39
item11	0.67	0.16	item11	0.98	−0.65
item12	0.99	1.16	item12	1.29	−0.13
item13	0.81	0.91	item13	1.19	−0.12
item14	0.42	2.79	item14	0.53	0.55
item15	0.69	−0.25	item15	0.76	−1.43
item16	0.93	−0.37	item16	0.70	−1.00
item19	0.48	−0.07	item19	0.61	−0.77
item20	1.29	−1.04	item20	1.21	−1.75
item21	0.82	1.01	item21	0.86	0.01
item22	0.71	−0.44	item22	0.76	−1.36
item23	0.68	−0.97	item23	1.03	−1.22
item24	0.61	1.51	item24	0.84	0.14
item25	1.76	−0.79	item25	1.17	−1.59
item26	0.40	2.54	item26	0.80	0.56
item27	0.47	0.12	item27	0.68	−1.06
item28	0.77	−0.54	item28	1.53	−1.00
item29	0.93	0.50	item29	1.06	−0.52
item30	1.32	0.08	item30	1.41	−0.62
item31	0.78	0.28	item31	0.88	−0.47
item32	1.34	−0.94	item32	1.32	−1.66
item33	1.19	−0.80	item33	1.34	−1.65
item34	0.47	2.60	item34	0.83	0.57
item35	1.04	−0.45	item35	1.18	−1.01
item36	0.53	0.41	item36	0.43	−0.56
item37	0.92	1.49	item37	0.78	0.51
item38	1.26	−2.01	item38	0.67	−4.47
item39	1.06	−0.39	item39	1.03	−1.06
item40	0.59	0.57	item40	0.64	−1.01
item41	0.52	−4.81	item41	0.43	−5.78
item42	1.24	0.03	item42	1.34	−1.05
item43	1.03	−0.94	item43	1.33	−1.65
item44	0.80	0.11	item44	1.29	−0.83
item45	1.28	−0.46	item45	1.58	−1.24
item46	1.15	−0.02	item46	1.43	−0.95
item47	0.76	0.32	item47	0.78	−0.89
item48	1.10	−0.46	item48	1.38	−1.13
item49	1.50	−1.03	item49	1.14	−1.78
item50	1.22	−0.55	item50	0.70	−2.02
item51	1.28	−0.62	item51	1.04	−1.90
item52	0.78	0.28	item52	0.81	−0.35
item53	0.95	0.52	item53	1.26	−0.13
item54	1.72	0.54	item54	1.39	−0.43
item55	0.80	−0.83	item55	1.43	−1.53
item56	1.26	−1.07	item56	1.15	−1.96
item57	1.71	−0.66	item57	1.36	−1.34
item58	1.35	−1.09	item58	1.39	−1.55
item59	0.69	0.95	item59	0.43	−0.97
item60	0.73	0.02	item60	1.19	−0.87
item61	1.54	0.04	item61	1.02	−1.29
item62	1.41	−1.11	item62	1.29	−1.86
item63	0.86	0.26	item63	0.86	−0.76
item65	1.41	−0.68	item65	1.62	−1.30
item67	0.98	−1.23	item67	1.25	−1.65
item68	0.34	−9.70	item68	0.79	−4.25
item69	1.93	−0.56	item69	1.21	−1.68
item70	1.31	−1.36	item70	1.22	−1.74
item71	1.31	−1.31	item71	1.54	−2.06
item72	1.01	−0.33	item72	1.30	−1.46
item73	1.35	0.04	item73	1.68	−0.75
item74	0.87	−0.16	item74	1.14	−0.92
item75	0.58	−0.35	item75	0.96	−1.23
item76	0.54	0.71	item76	0.86	−0.36
item78	0.99	0.44	item78	1.21	−0.37
item79	0.87	0.33	item79	1.05	−0.85
item80	1.24	−0.34	item80	1.36	−1.00

Note. *a* = *discrimination parameter*. The higher a value, the more sensitive it is to differences in ability; *a* < 0.65 = Low discrimination, 0.65 ≤ *a* < 1.35 = Moderate discrimination, 1.35 ≤ *a* < 1.70 = High discrimination, and *a* ≥ 1.70 = Very high discrimination (Baker, 2001); *b* = *item difficulty*. Higher *b* values correspond to items being more difficult; *b* < −2 = Very easy, −2 ≤ *b* < −1 = Easy, −1 ≤ *b* ≤ 1 = Medium difficulty, 1 < *b* ≤ 2 = Hard, *b* > 2 = Very hard (Baker, 2001).

**Table 4:** Summary of the mixed-effects linear regression model predicting Response Time (RT) as a function of Accuracy, Rest Score, Item Difficulty, and Country (with interactions).

	Log Response Time			
*Predictors*	Estimates	CI	*t*	*p*
(Intercept)	−0.591(0.190)	−0.963–−0.219	−3.114	0.002
Age	0.008(0.004)	0.001–0.015	2.151	0.031
Gender [Male]	0.012(0.041)	−0.067–0.092	0.305	0.760
Country [UK]	0.658(0.228)	0.211–1.106	2.882	0.004
Accuracy	1.427(0.142)	1.149–1.705	10.067	<0.001
rest score	0.035(0.003)	0.029–0.042	10.181	<0.001
item difficulty	0.338(0.322)	−0.293–0.968	1.050	0.294
Country [UK] × Accuracy	−0.613(0.193)	−0.992–−0.234	−3.170	0.002
Country [UK] × rest score	−0.014(0.005)	−0.024–−0.004	−2.741	0.006
Country [UK] × item difficulty	−0.689(0.385)	−1.443–0.066	−1.790	0.073
Accuracy × rest score	−0.026(0.003)	−0.032–−0.020	−8.701	<0.001
Accuracy × item difficulty	−0.825(0.439)	−1.685–0.036	−1.879	0.060
rest score × item difficulty	0.031(0.007)	0.017–0.044	4.507	<0.001
(Country [UK] × Accuracy) × rest score	0.008(0.005)	−0.000–0.017	1.874	0.061
(Country [UK] × Accuracy) × item difficulty	0.429(0.514)	−0.579–1.436	0.834	0.404
(Country [UK] × rest score) × item difficulty	0.007(0.009)	−0.011–0.024	0.758	0.448
(Accuracy × rest score) × item difficulty	0.026(0.009)	0.009–0.043	2.973	0.003
(Country [UK] × Accuracy × rest score) × item difficulty	−0.008(0.011)	−0.029–0.013	−0.734	0.463
**Random Effects**				
σ^2^	0.64			
τ_00 ParticipantID_	0.17			
ICC	0.21			
N _ParticipantID_	432			
Observations	31,322			
Marginal R^2^/Conditional R^2^	0.28/0.43			

Note. Log(ResponseTime) ~ Age + Gender + Country * Accuracy * rest_score * item_difficulty + (1|ParticipantID). CI = 95% Confidence Interval for the estimates.

## Data Availability

Anonymized data, analysis code, and the Supplementary Materials have been uploaded to the Open Science Framework: https://osf.io/db8fz/overview?view_only=a32e76d943a64672a9ec182a79fdffe5 (accessed on 20 January 2026).

## Note

1 To ensure data quality, we applied trial filtering standards based on those used in Chierchia et al. (2019). However, given our aim to conduct cross-cultural comparisons at the participant level, we modified the filtering approach. Instead of excluding individual trials with response times below 2.5 s, we excluded participants whose average response time across trials was below 2.5 s. This adjustment allowed us to normalize the number of trials across participants for cross-cultural comparison, thereby better capturing overall performance for each individual and ensuring that the data remain comparable across countries in subsequent analyses.

## Supplementary Materials

The following supporting information can be downloaded at https://www.mdpi.com/article/10.3390/jintelligence14070117/s1; Section S1: Assumption Checks for LMM; Section S2: Item Functioning Results; Section S3: Differential Item Functioning Analysis; Section S4: Model Comparison and Regression Coefficients for Speed–Accuracy Functions; Section S5: IES Calculation and Correlations Between Accuracy, Response Time and IES; Section S6: Lord’s Chi-Square Test for Differential Item Functioning (DIF) Across Groups; Section S7: Representative Item Characteristic Curves (ICCs) for Chinese (CN) and UK Samples; Section S8: Scatter Plot of Item Discrimination (a) vs. Item Difficulty (b) for Chinese (CN) and UK Samples; Section S9: Interaction Plots Derived from the LMM; Section S10: Two-Way ANOVA Results for the Effects of Country and Gender on Accuracy, Response Time, and Inverse Efficiency.
