# Supplementary material for: Task Engagement in Matrix Reasoning Performance: A Cross-Cultural Investigation in China and the United Kingdom
Source: J Intell. 2026 Jun 25;14(7):117. doi: 10.3390/jintelligence14070117 (PMC13412592; doi:10.3390/jintelligence14070117)
Supplement: Supplementary file 1 [file jintelligence-14-00117-s001.zip › jintelligence-4197311-supplementary.pdf]

Section S1 Assumption Checks for LMM

To ensure the statistical validity of using LMM, we performed several assumption checks using the *performance* (Lüdecke et al., 2021) and *see* (Lüdecke et al., 2020) packages. First, the linearity assumption was verified via residual-versus-fitted plots to ensure that the relationship between predictors and the outcome was appropriately captured by the linear functional form. Second, considering the skewed data on response time, following Zorowitz et al. (2023), we conducted a log-transformation to the response time data to mitigate the inherent right-skewness issue. Third, the homoscedasticity (homogeneity of variance) of residuals was assessed to confirm that the error variance remained stable across the range of predicted values. Visual inspections of the diagnostic plots indicated that these assumptions were well-satisfied.

The green reference lines in the residuals-versus-fitted and scale-location plots remained approximately horizontal and flat, confirming both linearity and homoscedasticity. Furthermore, the distribution of residuals closely followed the theoretical normal curve, demonstrating that the normality assumption held. While minor deviations were observed at the extremes of the fitted values, these are typical in trial-level behavioral data and do not compromise the integrity of the model estimates.

Section S1-Figure (a)

Assessment of Linearity: Residuals versus Fitted Values

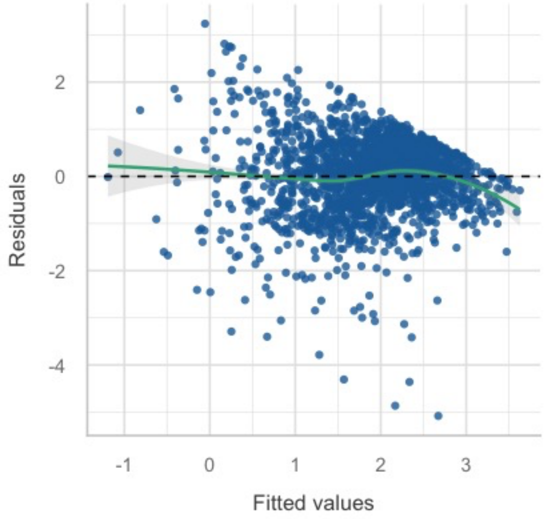

Note. This plot evaluates the linearity assumption of the mixed-effects model by plotting the residuals against the predicted (fitted) values. For a well-specified model, the residuals should be randomly dispersed around the horizontal dashed line at zero, with the green trend line remaining relatively flat. The observed distribution indicates that the linear relationship between the predictors and the log-transformed response time is adequately captured, with no major systematic non-linear patterns detected.

Section S1-Figure (b)

Assessment of Homoscedasticity: Scale-Location Plot of Standardized Residuals

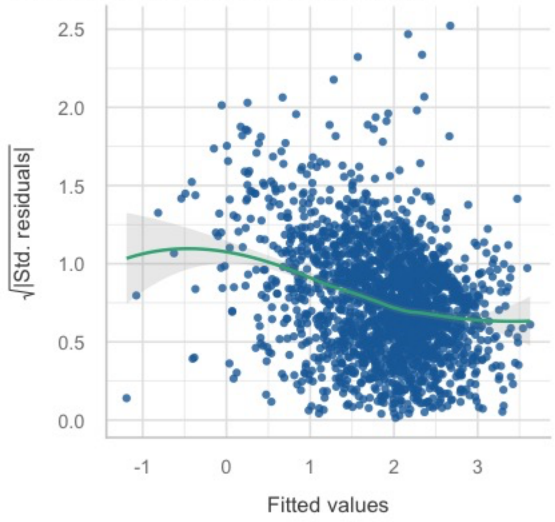

Note. This figure displays the Scale-Location plot (also known as the Spread-Level plot) used to assess the homogeneity of variance (homoscedasticity) for the linear mixed-effects model. The x-axis represents the fitted values from the model, while the y-axis shows the square root of the absolute standardized residuals. While a visually mild heteroscedasticity can be observed from the slight curvature of the green reference line, it remains within an acceptable range and does not jeopardize statistical inference. Such mild heteroscedasticity may increase standard errors, they do not necessarily invalidate regression estimates or significance testing when model stability remains intact (O'Brien, 2007). Therefore, the model estimates and inferential statistics remained robust following the log-transformation of the Response Time data.

Section S1-Figure (c)

Density Plot of Residuals for the Normality Assessment

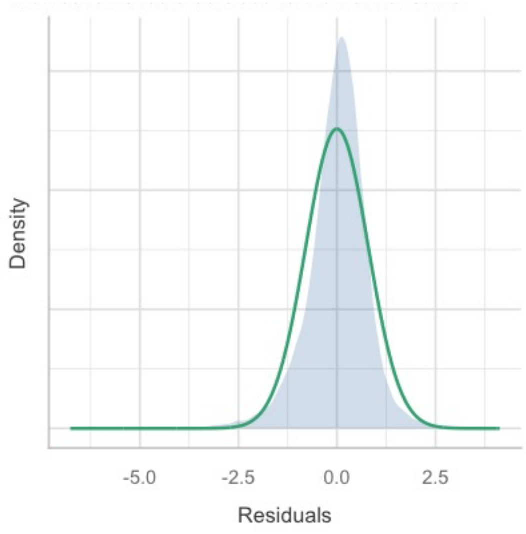

Note. This figure displays the density distribution of the model residuals (shaded area) compared against a theoretical normal distribution (solid green line). This visual diagnostic is used to verify the normality assumption of the linear mixed-effects

model. The close alignment between the observed residual density and the theoretical curve, centered around zero, indicates that the model residuals are approximately normally distributed. This supports the validity of the parameter estimates and the associated statistical inferences derived from the log-transformed response time data.

Section S2 Item Functioning Results

UK	S-X^2	df	p	CN	S-X^2	df	p
item1	5.59	15.00	.986	item1	18.37	13.00	.144
item2	17.06	12.00	.147	item2	3.31	11.00	.986
item3	7.76	8.00	.458	item3	1.47	2.00	.480
item4	19.12	18.00	.384	item4	3.45	5.00	.631
item5	9.67	11.00	.560	item5	1.93	5.00	.858
item6	30.27	30.00	.452	item6	29.64	31.00	.536
item7	8.47	14.00	.863	item7	1.91	5.00	.861
item8	29.80	20.00	.073	item8	11.10	9.00	.269
item9	17.31	20.00	.633	item9	12.47	13.00	.489
item10	41.83	32.00	.115	item10	25.76	24.00	.366
item11	44.76	39.00	.243	item11	25.28	30.00	.711
item12	22.46	28.00	.760	item12	27.29	25.00	.342
item13	37.93	33.00	.255	item13	29.27	26.00	.299
item14	24.99	31.00	.768	item14	29.58	30.00	.487
item15	29.90	37.00	.790	item15	42.82	30.00	.061
item16	39.63	34.00	.233	item16	34.28	32.00	.359
item17	21.39	31.00	.901	item17	44.67	29.00	.032
item18	22.98	33.00	.904	item18	31.66	26.00	.205
item19	51.47	39.00	.087	item19	22.14	34.00	.942
item20	10.94	25.00	.993	item20	14.99	18.00	.662
item21	32.59	33.00	.487	item21	32.66	30.00	.338
item22	37.36	36.00	.407	item22	26.19	32.00	.755
item23	37.18	33.00	.283	item23	16.18	27.00	.949
item24	38.89	34.00	.259	item24	30.12	30.00	.460
item25	36.71	24.00	.047	item25	25.08	21.00	.244
item26	31.09	33.00	.563	item26	22.79	25.00	.590
item27	44.99	40.00	.271	item27	30.73	32.00	.531
item28	40.67	35.00	.235	item28	15.62	24.00	.901
item29	38.81	33.00	.224	item29	32.07	30.00	.364
item30	25.30	31.00	.754	item30	34.68	27.00	.147
item31	37.59	37.00	.442	item31	23.09	30.00	.812
item32	32.88	26.00	.166	item32	21.35	17.00	.211
item33	39.73	30.00	.110	item33	21.90	18.00	.236
item34	40.53	29.00	.076	item34	28.19	25.00	.299
item35	31.22	31.00	.455	item35	25.92	27.00	.523
item36	34.65	39.00	.669	item36	20.97	34.00	.961
item37	19.63	26.00	.809	item37	27.25	27.00	.450
item38	23.80	17.00	.125	item38	5.19	9.00	.818
item39	27.99	33.00	.715	item39	32.60	29.00	.294

item40	51.99	39.00	.080	item40	37.82	34.00	.299
item41	15.06	13.00	.303	item41	7.54	12.00	.820
item42	35.98	32.00	.288	item42	28.31	24.00	.247
item43	25.91	30.00	.680	item43	29.03	17.00	.034
item44	30.32	37.00	.773	item44	31.39	26.00	.214
item45	34.60	30.00	.257	item45	17.74	21.00	.666
item46	31.77	33.00	.528	item46	30.61	26.00	.243
item47	31.48	39.00	.799	item47	28.51	31.00	.595
item48	26.75	31.00	.685	item48	38.04	24.00	.034
item49	22.76	21.00	.357	item49	16.89	20.00	.660
item50	27.71	30.00	.586	item50	22.60	26.00	.655
item51	50.94	30.00	.010	item51	34.85	20.00	.021
item52	49.89	38.00	.094	item52	45.07	32.00	.063
item53	33.59	33.00	.439	item53	21.99	26.00	.689
item54	22.39	25.00	.613	item54	39.36	28.00	.075
item55	32.76	33.00	.479	item55	19.64	17.00	.293
item56	22.97	25.00	.580	item56	20.38	18.00	.312
item57	40.34	26.00	.036	item57	27.96	23.00	.217
item58	17.61	21.00	.674	item58	16.62	17.00	.480
item59	36.19	35.00	.413	item59	36.39	34.00	.358
item60	34.87	37.00	.569	item60	24.15	28.00	.674
item61	21.41	29.00	.844	item61	31.03	27.00	.270
item62	21.04	21.00	.457	item62	11.74	17.00	.816
item63	30.17	36.00	.741	item63	44.20	31.00	.059
item64	48.45	35.00	.065	item64	31.65	31.00	.434
item65	24.28	28.00	.667	item65	15.87	18.00	.601
item66	38.13	34.00	.287	item66	37.55	29.00	.133
item67	35.15	27.00	.135	item67	19.02	18.00	.390
item68	8.92	5.00	.112	item68	4.82	6.00	.567
item69	24.66	25.00	.482	item69	26.50	19.00	.117
item70	26.84	22.00	.217	item70	12.58	18.00	.816
item71	25.41	22.00	.278	item71	15.99	11.00	.141
item72	24.24	34.00	.892	item72	18.07	21.00	.645
item73	22.30	32.00	.899	item73	30.96	23.00	.124
item74	46.03	36.00	.122	item74	32.26	27.00	.222
item75	49.45	38.00	.101	item75	42.81	29.00	.047
item76	57.29	40.00	.037	item76	31.39	31.00	.447
item77	18.79	27.00	.878	item77	23.72	26.00	.592
item78	39.40	33.00	.205	item78	27.60	28.00	.486
item79	40.38	36.00	.283	item79	32.01	31.00	.416
item80	18.83	30.00	.944	item80	28.34	26.00	.342

Section S3 Differential Item Functioning Analysis

Item	Chi-square	P-value
item1	0.42	.811
item2	7.06	.029
item3	1.82	.403
item4	1.29	.526
item5	0.40	.819
item6	12.28	.002
item7	0.52	.770
item8	0.15	.928
item9	5.09	.078
item10	0.27	.872
item11	0.58	.748
item12	1.02	.601
item13	1.84	.399
item14	2.22	.330
item15	1.54	.463
item16	7.31	.026
item17	9.43	.009
item18	9.95	.007
item19	1.04	.595
item20	3.79	.150
item21	3.27	.195
item22	2.35	.308
item23	2.16	.339
item24	0.82	.663
item25	9.17	.010
item26	0.23	.893
item27	0.42	.812
item28	1.58	.454
item29	3.25	.197
item30	4.76	.093
item31	3.78	.151
item32	4.47	.107
item33	2.04	.361
item34	0.11	.944
item35	6.24	.044
item36	3.70	.157
item37	4.98	.083

item38	5.38	.068
item39	4.04	.132
item40	1.77	.413
item41	0.54	.764
item42	3.28	.194
item43	0.26	.876
item44	0.85	.653
item45	1.24	.538
item46	1.68	.433
item47	2.56	.278
item48	2.14	.344
item49	6.06	.048
item50	8.68	.013
item51	5.95	.051
item52	5.50	.064
item53	5.82	.055
item54	8.80	.012
item55	0.15	.927
item56	1.97	.373
item57	8.09	.018
item58	5.15	.076
item59	6.19	.045
item60	0.46	.796
item61	11.19	.004
item62	1.25	.536
item63	3.31	.191
item64	2.93	.231
item65	1.73	.421
item66	10.75	.005
item67	1.95	.378
item68	0.11	.945
item69	9.53	.009
item70	5.06	.080
item71	1.18	.553
item72	1.99	.370
item73	2.68	.262
item74	1.24	.539
item75	0.01	.997
item76	0.06	.971
item77	11.41	.003
item78	3.47	.176
item79	0.59	.743
item80	4.95	.084

Section S4 Model Comparison and Regression Coefficients for Speed–Accuracy Functions

To formally evaluate whether the relationship between response time and performance accuracy differs across countries, we conducted an incremental model comparison. We compared a constrained model assuming a single, common speed–accuracy trade-off (SAT) function (Model 1: Accuracy ~ Speed) against an interaction model allowing group-specific intercepts and slopes (Model 2: Accuracy ~ Speed × Country). The incremental F-test demonstrates that allowing group-specific intercepts and slopes yields a significantly superior model fit ($F(2, 96) = 11.70$, $p < .001$), rejecting the account of a single, shared performance curve.

Section S4-Table (a)

ANOVA Table Comparing the Common SAT Model and the Country-Specific Interaction Model

Model Specification	Df	Sum of Sq	Residual Df	Residual Sum of Sq	F	p
Model1: Accuracy~ Response Time	—	—	98	0.295	—	—
Model 2: Accuracy~ Response Time × Country	2	0.058	96	0.237	11.698	<.001

Section S4-Table (b)

Regression Coefficients and Parameter Estimates for the Interaction Model (Model 2)

Predictors	Coefficient Estimate (B)	Standard Error (SE)	t-value	p

(Intercept) [Chinese Baseline]	0.472	0.031	15.010	<.001***
Response Time (s)	0.088	0.012	7.166	<.001***
Country [UK]	0.169	0.049	3.465	<.001***
Response Time × Country [UK]	-0.073	0.018	-4.110	<.001***

Note. Model Fit Metrics: Residual Standard Error = 0.0497 on 96 degrees of freedom; Multiple R^2 = .3567\$, Adjusted R^2 = .3366\$; Overall Model F(3, 96) = 17.75, $p < .001$. The baseline reference group for the factor levels is set to the Chinese (CN) cohort.

Section S5 IES Calculation and Correlations Between Accuracy, Response Time and IES

We recorded accuracy and median response times for correct trials at both the trial and sample levels as our primary metrics for data analysis. Additionally, we calculated the Inverse Efficiency Score (IES) by dividing the median response time by accuracy (Townsend, 1978).

$$\text{Inverse Efficiency Score (IES)} = \frac{Response\ time\ (in\ seconds, to\ make\ correct\ response)}{Accuracy\ (number\ correct\ response)}$$

To investigate potential informational overlap and the construct validity of these measures, we then conducted correlation analyses across the CN, UK, and merged samples. Across all three groups, a consistent and significant correlational pattern emerged between the performance metrics of Accuracy, Response Time, and IES.

Specifically, in the CN group, Accuracy was significantly and positively correlated with Response Time ($r = 0.60$, $p < .001$), suggesting a potential speed-accuracy trade-off. Additionally, Accuracy showed a significant negative correlation with IES ($r = -0.38$, $p < .001$), while Response Time was significantly and positively associated with IES ($r = 0.47$, $p < .001$). For the UK sample, Accuracy and Response Time were significantly and positively correlated ($r = 0.61$, $p < .001$). Accuracy was negatively correlated with IES, though the relationship was weaker than in the CN group ($r = -0.16$, $p < .05$). Conversely, Response Time showed a strong, significant positive correlation with IES ($r = 0.65$, $p < .001$). In the merged analysis, all

correlations remained highly significant. Accuracy and Response Time exhibited a strong positive correlation (r = 0.67, $p < .001$). Furthermore, Accuracy was negatively correlated with IES (r = -0.19, $p < .001$), and Response Time was positively correlated with IES (r = 0.56, $p < .001$).

Section S5-Table(a)

Correlations Between Accuracy, Response Time and IES across different sample groups

Sample	Variable	1. ACC	2. RT	3. IES
CN (N=235)	1. Accuracy	-		
	2. Response Time	0.60***	-	
	3. IES	-0.38***	0.47***	-
UK (N=222)	1. Accuracy	-		
	2. Response Time	0.61***	-	
	3. IES	-0.16*	0.65***	-
Pooled (N=457)	1. Accuracy	-		
	2. Response Time	0.67***	-	
	3. IES	-0.19***	0.56***	-

Section S6 ***Lord's Chi-Square Test for Differential Item Functioning (DIF) Across Groups***

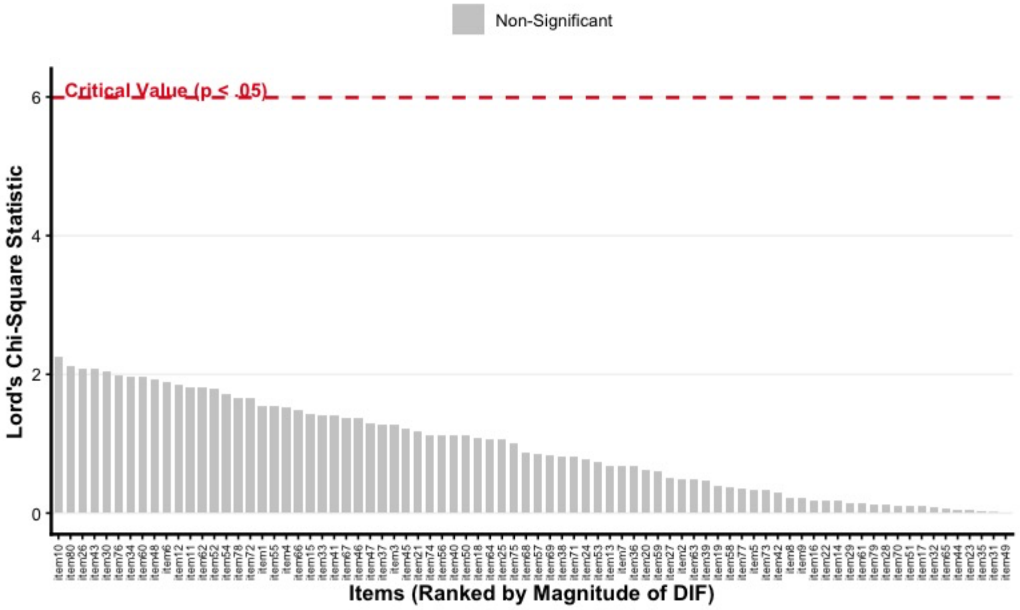

Note. The figure displays the Lord's Chi-square statistics for all 80 items comparing the UK and Chinese samples based on the 2PL IRT model. The horizontal red dashed line represents the critical value ($\chi^2 = 5.99$, $df = 2$, $p = .05$). Items exceeding this threshold are traditionally flagged for significant DIF. As shown, all items remain below the formal significance threshold, indicating that the item parameters (discrimination and difficulty) are generally invariant across the two cultural contexts. Despite this statistical consistency, a subset of items (items 6, 17, 18, 61, 64, 66, and 77) was subsequently removed to further optimize model fit and ensure the highest degree of measurement precision for the final cross-cultural comparison. Items are ranked from left to right by the magnitude of their Chi-square values.

Section S7 ***Representative Item Characteristic Curves (ICCs) for Chinese (CN) and UK Samples.***

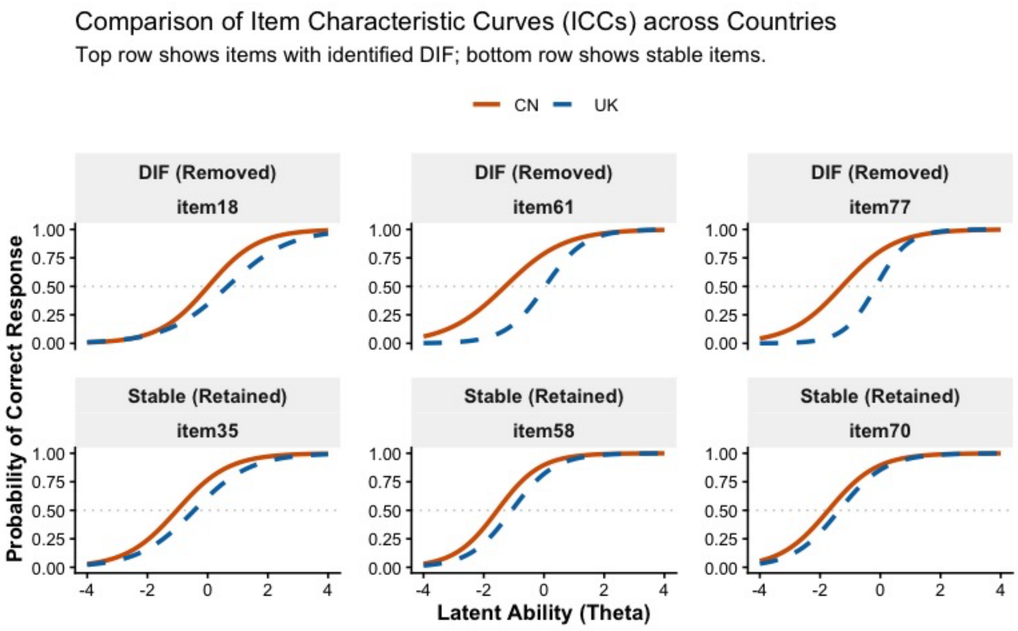

Note. The figure displays the ICCs for four representative items calculated using the Two-Parameter Logistic (2PL) model. Theta = ability estimate. The dashed horizontal line indicates a 0.5 probability level, corresponding to the item difficulty parameter. Distinct gaps between the curves for the CN (orange) and UK (blue) samples illustrate Differential Item Functioning (DIF). The top row displays three of the removed items (e.g., items 18, 61, 77) that showed the high degree of divergence between groups, these items were excluded from the final analysis to further enhance measurement invariance and model fit. The bottom row showcases stable items (e.g., items 35, 58, 70) where the probability traces demonstrate high convergence across the latent ability spectrum (theta), providing evidence of cross-cultural measurement equivalence for the retained item pool. The figure presents the ICCs for representative

items based on the 2PL IRT model for the CN (solid line) and UK (dashed line) samples. The top row displays items (e.g., items 18, 61, 77) that showed the highest degree of divergence between groups; although their Lord's Chi-square values did not reach formal significance, these items were excluded from the final analysis to further enhance measurement invariance and model fit. The bottom row showcases stable items (e.g., items 35, 58, 70) where the probability traces demonstrate high convergence across the latent ability spectrum (θ), providing evidence of cross-cultural measurement equivalence for the retained item pool.

Section S8 ***Scatter Plot of Item Discrimination (a) vs. Item Difficulty (b) for Chinese (CN) and UK Samples.***

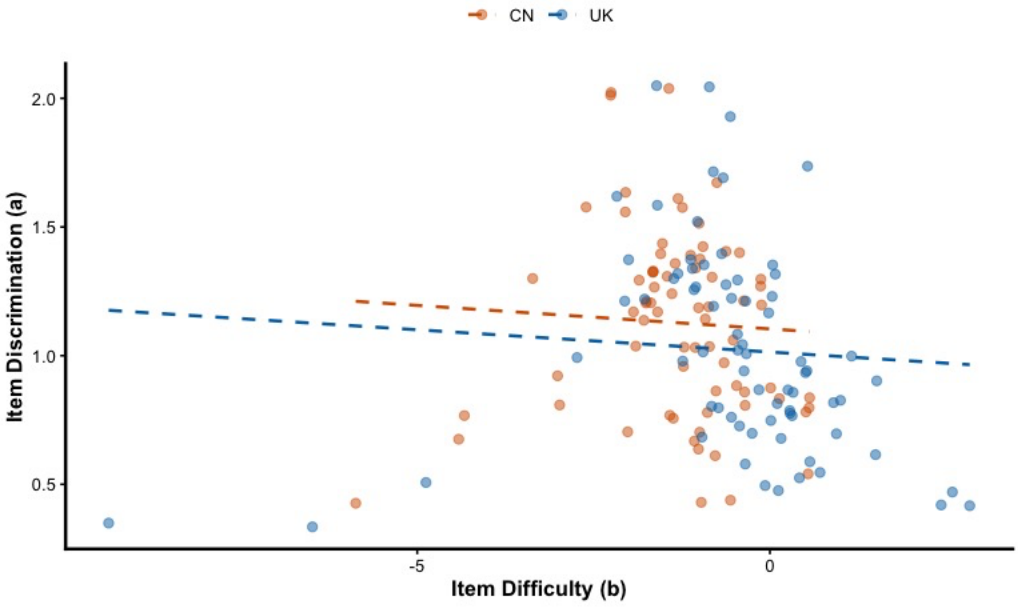

Note. This figure illustrates the calibration of item parameters within the Two-Parameter Logistic (2PL) model framework for the Chinese (CN, orange) and United Kingdom (UK, blue) samples. The horizontal axis represents the item difficulty parameter (*b*), while the vertical axis represents the item discrimination parameter (*a*). Each point corresponds to an individual item in the assessment. The dashed lines represent linear regression trends for each group. The substantial overlap between the two distributions suggests that the items generally function consistently across both cultural contexts. However, the UK sample shows a broader distribution toward higher difficulty levels ($b > 0$), whereas the CN sample contains a higher density of items at lower difficulty levels ($b < -2$), aligning with observed differences in group performance.

Section S9 Interaction Plots Derived from the LMM

Section S9-Figure (a)

Predicted Response Times as a Function of Accuracy and Country.

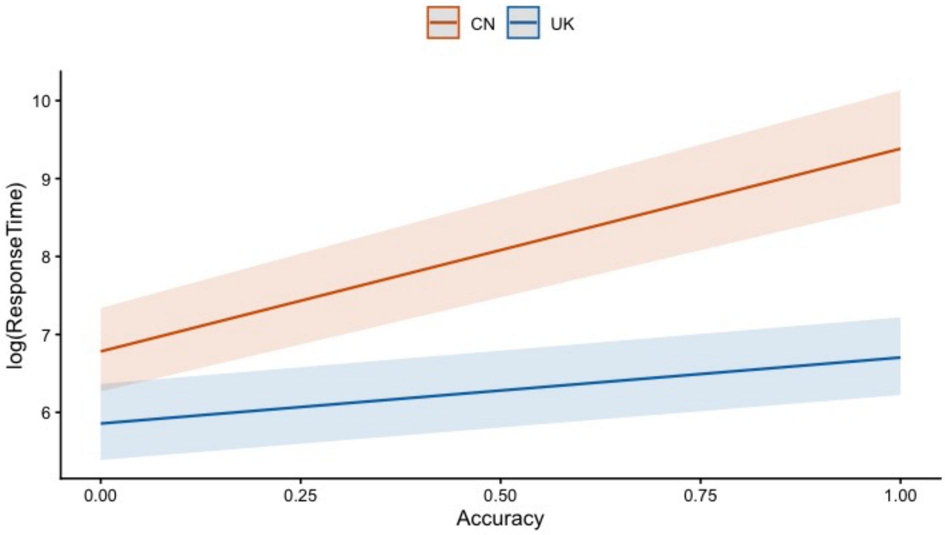

Note. The figure illustrates the predicted response times (RT), back-transformed from the log scale to milliseconds, for incorrect (0.00) versus correct (1.00) responses across the Chinese (CN) and UK samples. Shaded areas represent the 95% confidence intervals.

Section S9-Figure (b)

Predicted Response Times as a Function of Rest Score and Country.

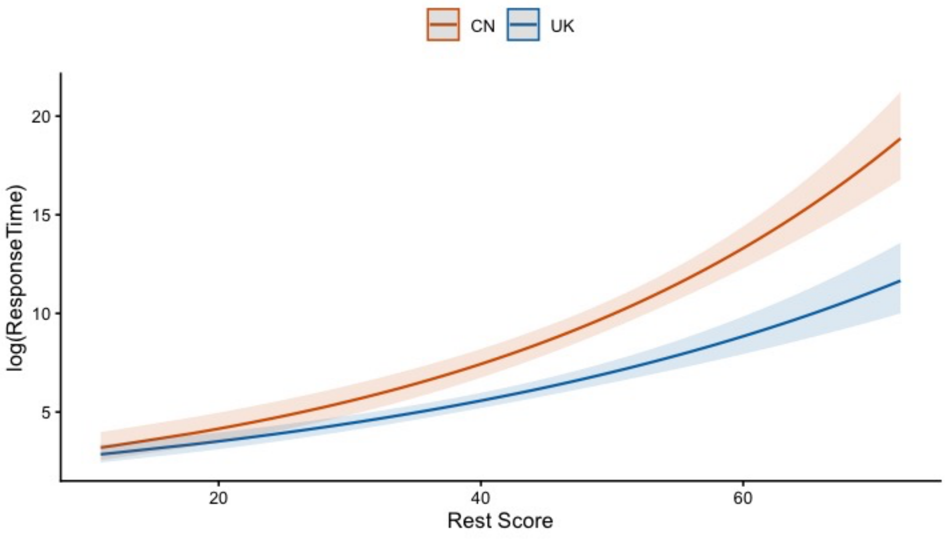

Note. This plot illustrates the relationship between participants' latent ability (rest scores) and their predicted response times (RT), back-transformed to milliseconds. Shaded areas indicate 95% confidence intervals.

Section S10 Two-Way ANOVA Results for the Effects of Country and Gender on Accuracy, Response Time, and Inverse Efficiency

We found statistically significant main effects of Country, Gender, and a significant interaction effect between Country and Gender on accuracy, response time, and efficiency (see Table 4). For accuracy, there was a significant difference between countries (China > UK), but no significant effect of Gender or the Country × Gender interaction.

In terms of response time, Country had a significant effect, with participants from the UK responding faster than those from China, but there was no observed significant effect of gender. The Country × Gender interaction was significant, with pairwise comparisons showing that both males and females from China outperformed both males and females from the UK, and that the UK males also performed better than UK females. It was also worth noticing that unlike the gender difference in response time observed in the UK sample, participants in the Chinese sample did not appear to show a comparable gender difference in response time. This pattern warrants further investigation.

For inverse efficiency score, Country had a significant effect (China > UK), indicating that the UK was more efficient than China. Gender also influenced inverse efficiency (male > female), with females being more efficient than males. However, the interaction between Country and Gender was not significant.

These findings suggest that China outperforms the UK in terms of accuracy, but that the UK outperforms China in terms of response time and efficiency, and that females generally perform better than males on efficiency.

Section S10-Table (a).

Country × Gender ANOVA: Accuracy, Response Time, Efficiency

Parameter	Factor	*df*	*SS*	*MS*	*F*	*p*	*Tukey HSD*
Accuracy	Country	1.00	2.43	2.43	82.60	<.001	China > UK
	Gender	1.00	0.00	0.00	0.13	.724	
	Country x Gender	1.00	0.02	0.02	0.64	.424	
	Residuals	428.00	12.61	0.03			
Response Time	Country	1.00	828.00	828.05	88.90	<.001	China > UK
	Gender	1.00	29.90	29.95	3.22	.074	
	Country x Gender	1.00	44.40	44.36	4.76	.030	China Female > UK Female; China Female > UK Male; China Male > UK Female;

							UK Male > UK Female; China Male > UK Male
	Residuals	428.00	3986.50	9.31			
Inverse Efficiency	Country	1.00	94.00	93.99	4.90	.027	China > UK
	Gender	1.00	110.90	110.93	5.79	.017	Male >Female
	Country x Gender	1.00	61.90	61.87	3.23	.073	
	Residuals	428.00	8203.80	19.17			
